# Anti-*Bordetella bronchiseptica* effects of targeted bacteriophages via microbiome and metabolic mediated mechanisms

**DOI:** 10.1038/s41598-023-49248-1

**Published:** 2023-12-08

**Authors:** Abdolreza Hosseindoust, YoHan Choi, SangHun Ha, Habeeb Tajudeen, JunYoung Mun, Elick Kinara, YoungIn Kim, JinSoo Kim

**Affiliations:** 1 Department of Animal Industry Convergence, Kangwon National University, Chuncheon, 24341 Republic of Korea; 2 Swine Science Division, National Institute of Animal Science, Rural Development Administration, Cheonan, 31000 Republic of Korea; 3 CTC Bio, Inc., Seoul, 138-858 Republic of Korea

**Keywords:** Biological techniques, Molecular biology, Physiology, Diseases

## Abstract

*Bordetella bronchiseptica* poses a significant challenge in the context of respiratory infections, particularly in weanling pigs. In this study, we investigated the impact of a novel targeted bacteriophage in controlling *B. bronchiseptica* challenge (BBC) in an experimental design involving five distinct treatment groups: NC (no challenge), PC (BBC challenge), BF (10^8^ pfu bacteriophage/kg diet + BBC), BN (2 × 10^7^ pfu/day bacteriophage by nasal spray + BBC), and AT (antibiotic + BBC). The experiment was conducted for 2 weeks. The highest turbinate score was observed in the PC. The BF treatment showed higher plasma IL (interleukine)-1β and IL-6 compared with the BN and AT treatments. Plasma concentrations of IL-1β were increased in the BF pigs compared with the BN, AT, and NC. Among the BBC groups, the PC treatment exhibited a higher abundance of *Staphylococcus. aureus* and *B. bronchiseptica* in the lung. A lower *S. aureus*, *Streptococcus. suis*, and *B. bronchiseptica* colonization was detected in the AT compared with the BF and BN treatments. The BF showed lower plasma zonulin compared with the BN and AT. A higher plasma concentration of superoxide dismutase was observed in the BF and AT compared with PC and BN. The BN influenced the glycine, serine-threonine metabolism; glycerolipid metabolism; glyoxylate-dicarboxylate metabolism; and arachidonic acid metabolism compared with the NC. In conclusion, nasal-sprayed bacteriophage effectively controlled *B. bronchiseptica* infection, however, their efficiency was lower than the antibiotic.

## Introduction

*Bordetella bronchiseptica* species are pathogenic bacteria with intense influence on the respiratory tract of a wide range of animals from farm animals to pets, and rabbits, with coughing, sneezing, enlarged lymph, and severe pneumonia symptoms^1,2^. The bacteria spread to the nasal epithelial lining by the airborne channel, leading to chronic infections^3^. Lung infection is a severe disease caused by *B. bronchiseptica*, particularly in intensive piggery systems*,* which was the main mortality reason in weanling pigs before introducing vaccines^4,5^. Currently, vaccination against *B. bronchiseptica* is a routine procedure for avoiding illnesses in animals^6^. Despite the development of several vaccination techniques, the existence of immunity for a long duration is being doubted. Outbreaks of *B. bronchiseptica* have occurred frequently in swine farms with considerable atrophic rhinitis losses^5^*.* Moreover, in 2022, there was a significant increase in canine infections caused by *B. bronchiseptica* and canine influenza due to increased human travel after the relaxation of the COVID-19 pandemic in the overcrowding of dogs and cats to be housed in kennels and daycare facilities. *B. bronchiseptica* is a mammalian-specific disease with systemic infection starting from the lung and gradually affecting the other organs^1,7^. The immune response to eliminate *B. bronchiseptica* infection is not fully understood, however, it was reported that this bacteria causes pigs more vulnerable to being infected by several other viral and opportunistic bacterial diseases^8–10^. In order to decrease the economic losses, it is crucial to control *B. bronchiseptica* infection at the initial stages in the lung. Lung infection has been successfully controlled by improved hygiene, antibiotic treatment, and vaccinations^1,11^. Additionally, the non-selective selection of antibiotics disrupts the intestinal microbiota and eradicates a number of beneficial bacteria that may increase the occurrence of lung infection^11^. Alternative methods of treating *B. bronchiseptica* infections are also necessary due to the emergence and spread of antibiotic-resistant *B. bronchiseptica* strains^12^. In this regard, the focus of attention has switched to the investigation of fresh preventative measures to reduce the prevalence of pneumonia in pigs.

The use of non-antibiotic agents can significantly limit the horizontal spread of *B. bronchiseptica* in farms, although antibiotics have long been a part of an efficient controlling approach for *Bordetella* spp. in swine farms^2^. The epidemiology of *B. bronchiseptica* infection relies on horizontal transmission since some infected pigs become long-term, asymptomatic carriers of *B. bronchiseptica* and spread it to healthy pigs^13^. The relevance of using bacteriophages to remove infections from challenged pigs is increased by their specificity in omitting targeted pathogens^14–16^. In addition, bacteriophages could be administered in a long term before and after the occurrence of swine pneumonia to limit the initial spread of disease. Semi-pH-stable bacteriophages with the capability of surviving in the respiratory tract were produced owing to the ongoing advancements in manufacturing technology. To lessen the severity of pneumonia in the gastrointestinal system, bacteriophages can be administered singly or in combination.

The treatment with bacteriophages or antibiotics can affect the body metabolic pathways due to the change in the microbiome and immune status^17^, potentially serving as a marker for assessing pigs nutritional requirements. Blood functional metabolomics is the study of small molecule metabolites in blood, which can provide insights into the metabolic processes and functional states of various biological systems, including the immune system^18–20^. The metabolites in the blood can reflect changes in the immune system, and their levels can be influenced by nutrition and diet^21^. In terms of feed requirements, diet, and nutrition can impact the levels of certain metabolites in blood and can be detected by metabolomics programming^22–24^. Exogenous addition of some nutrients in the diet provided to pigs during the infection can promote the immune system and growth performance. To our current understanding, this is the first pH-stable bacteriophage with specific targeting capabilities against *B. bronchiseptica* in pigs, with potential applicability across canine and other mammalian species. As bacteriophages can be transferred from dietary intake to the respiratory tract through inhalation, we tried to test the addition of bacteriophages in the diet in order to find an easier solution to protect or cure animals on a large scale. The purpose of this study was to assess how anti-*Bordetella* bacteriophages in diet or nasal spray can control *B. bronchiseptica* disease, immunological function, and disease prevention in pigs exposed to *B. bronchiseptica*.

## Results

### Turbinate score

The turbinate score result is shown in Table 1. The pigs in the PC group showed the highest scroll number. The scroll number of BF pigs was higher than the AT and NC. The septal number was higher in the PC pigs compared with BN, AT, and NC. The total turbinate score result showed the highest number in the PC. There was no difference between the BF and BN, however, the turbinate score of pigs in the AT treatment was lower than the BF treatment.

**Table 1:** Effects of bacteriophage on turbinate score of weanling piglets challenged with *Bordetella bronchiseptica* (BBC)*.*

Treatment^1^	NC	Challenge (*Bordetella bronchiseptica*)				SEM^2^	*p*-value
		PC	BF	BN	AT		
Turbinate score							
Scroll (0–16)	0.83^c^	5.50^a^	2.83^b^	1.50^bc^	1.00^c^	0.36	< 0.001
Septal (0–2)	0.00^b^	0.67^a^	0.33^ab^	0.00^b^	0.00^b^	0.28	0.015
Total (0–18)	0.83^c^	6.17^a^	3.17^b^	1.50^bc^	1.00^c^	0.42	< 0.001

^1^NC, control group; PC, with BBC; BF, bacteriophage in feed (10^8^ pfu /kg diet) + BBC; BN, bacteriophage nasal spray (2 × 10^7^ pfu) + BBC; AT, 1 g florfenicol/kg + BBC.

^2^SEM, standard error of means.

^a–c^means with different superscripts in the same row differ significantly (*p* < 0.05).

### Inflammatory cytokines

The concentration of plasma IL (interleukine)-1β, IL-6, and tumor necrosis factor-α (TNF-α) was increased in PC pigs compared with the NC 7 days after the *Bordetella. bronchiseptica* challenge (BBC) (Table 2). The BF treatment showed higher plasma IL-1β and IL-6 compared with the BN and AT treatments. Similarly, compared with the AT, the plasma concentration of TNF-α was markedly increased in BF pigs at day 7. At day 14, the PC pigs showed the highest IL-1β, IL-6, and TNF-α compared with the NC, BF, BN, and AT pigs. plasma concentrations of IL-1β were markedly increased in the BF pigs compared with the BN, AT, and NC, whereas IL-1β concentration was the lowest in NC. The plasma IL-6 and TNF-α levels were higher in the BF pigs compared with the BN, AT, and NC. A higher plasma concentration of IL-6 was observed in the BN treatment compared with the NC and AT, however, no difference was detected in the concentration of TNF-α between the NC and AT treatments.

**Table 2:** Effects of inflammatory cytokines on bacteriophage of weanling piglets challenged with *Bordetella bronchiseptica* (BBC)*.*

Treatment^1^	NC	Challenge (*Bordetella bronchiseptica*)				SEM^2^	*p*-value
		PC	BF	BN	AT		
Phase 1 (d 7)							
IL-1β	25.1^d^	139.0^a^	121.1^b^	104.6^c^	93.3^c^	3.82	< 0.001
IL-6	127.6^e^	323.8^a^	252.6^b^	199.3^c^	161.0^d^	8.32	< 0.001
TNF-α	189.4^d^	289.7^a^	230.8^b^	220.5^bc^	210.1^c^	8.79	< 0.001
Phase 2 (d 14)							
IL-1β	37.8^d^	190.3^a^	113.3^b^	97.7^c^	88.7^c^	4.09	< 0.001
IL-6	117.4^e^	303.1^a^	224.3^b^	179.8^c^	141.2^d^	8.44	< 0.001
TNF-α	251.4^c^	324.2^a^	287.9^b^	257.7^c^	241.8^c^	10.9	< 0.001

^1^NC, control group; PC, with BBC; BF, bacteriophage in feed (10^8^ pfu /kg diet) + BBC; BN, bacteriophage nasal spray (2 × 10^7^ pfu) + BBC; AT, 1 g florfenicol/kg + BBC.

^2^SEM, standard error of means.

^a–d^means with different superscripts in the same row differ significantly(p < 0.05).

### Opportunistic bacteria

**Figure 1:**
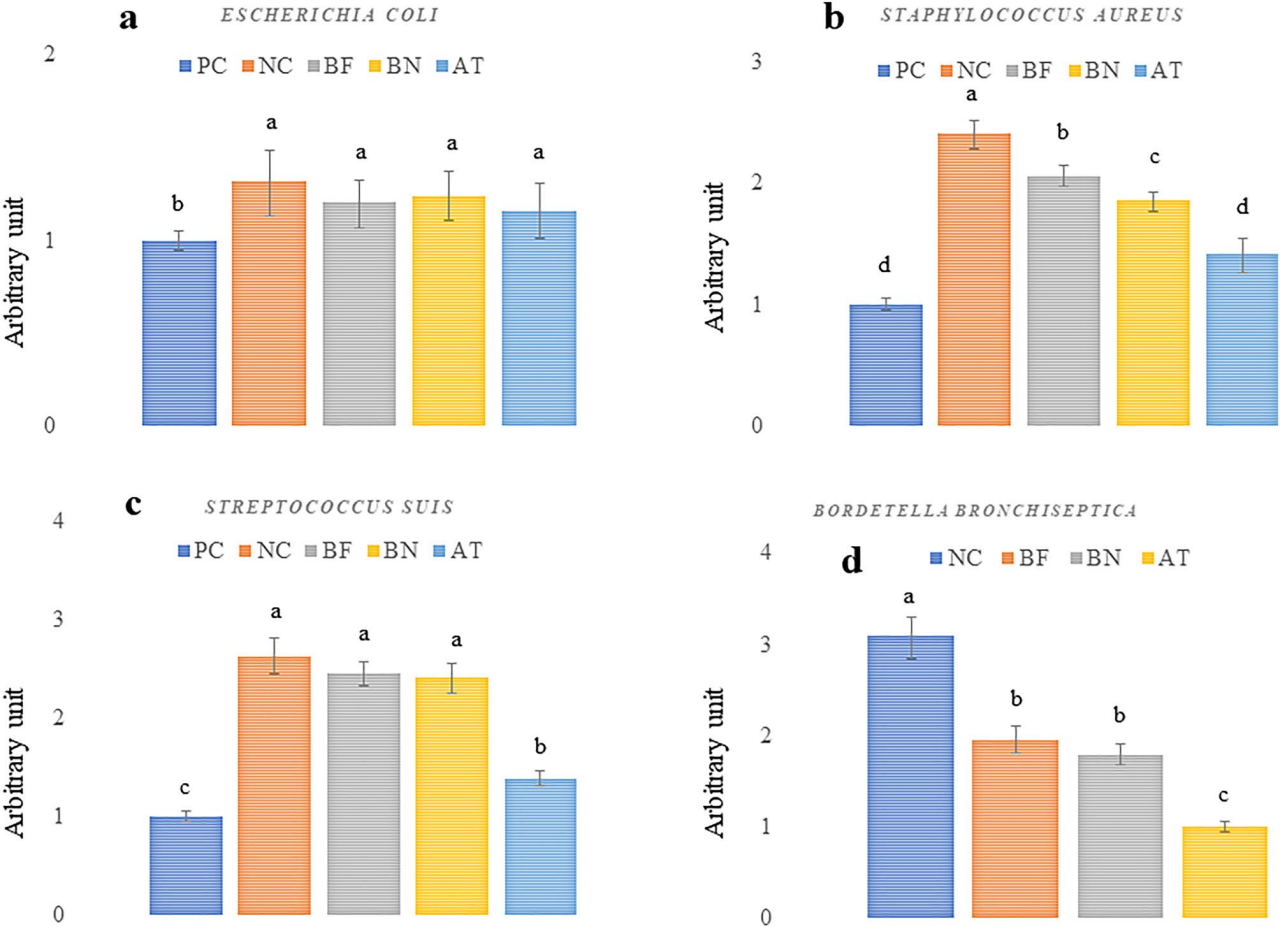
Effects of bacteriophage and *Bordetella bronchiseptica* challenge (BBC) on the relative abundance in lung bacteria including *Escherichia coli* (**a**), *Staphylococcus aureus* (**b**), *Streptococcus suis* (**c**), and *Bordetella bronchiseptica* (**d**) of weanling piglets in the NC, control group; PC, with BBC; BF, bacteriophage in feed (10^8^ pfu /kg diet) + BBC; BN, bacteriophage nasal spray (2 × 10^7^ pfu) + BBC; AT, 1 g florfenicol/kg + BBC. ^1^NC, control group; NC + BBC; BF, bacteriophage in feed (108 pfu /kg diet) + BBC; BN, bacteriophage nasal spray (2 × 107 pfu) + BBC; AT, 1 g florfenicol/kg + BBC.

The relative abundance of opportunistic bacteria is shown in Fig. 1. The pigs in the NC group exhibited a decreased population of *E. coli*, *S. aureus*, and *S. suis* in the lung compared with the BBC groups. Among the BBC groups, the PC treatment exhibited a higher abundance of *S. aureus* and *B. bronchiseptica* in the lung. At d 14 after the BBC groups, a lower *S. aureus*, *S. suis*, and *B. bronchiseptica* population were detected in the AT group compared with the BF and BN pigs. The control treatment was not infected and showed negative results for *B. bronchiseptica* detection throughout the experiment. Supplementation of bacteriophage in diet and nasal spray had no difference on the population of *E. coli*, *S. suis,* and *B. bronchiseptica*, however, the abundance of *S. aureus* significantly decreased in the BN treatment compared with the BF treatment.

### Zonolin, lipocaline-2, lipopolysaccharide, lipopolysaccharide-binding protein, superoxide dismutase, and malondialdehyde

**Figure 2:**
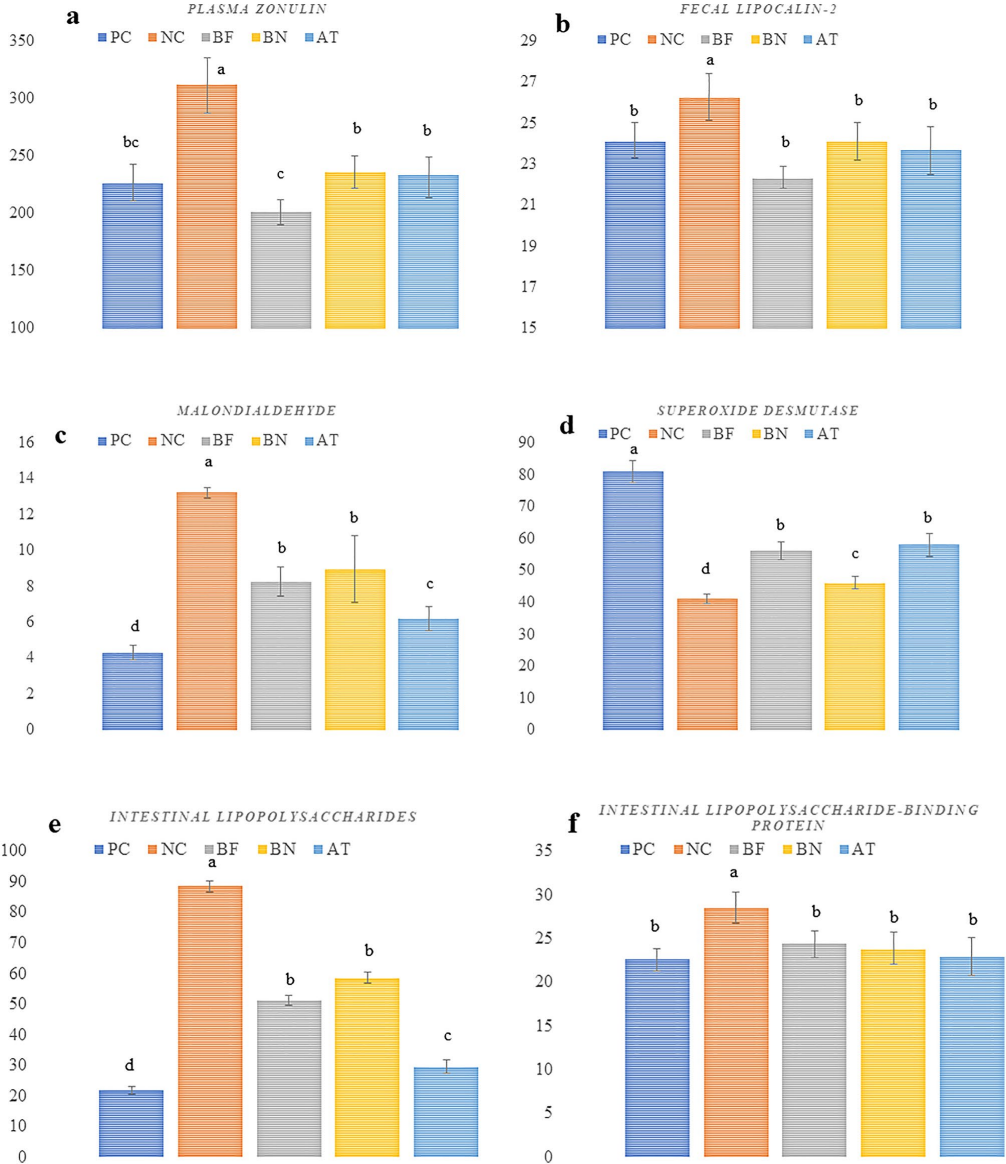
Effects of bacteriophage and *Bordetella bronchiseptica* challenge (BBC) on plasma zonulin, maloniadehyde, superoxide dismutase, fecal lipocaline-2, and intestinal lipopolysachrides and lipopolysachride binding protein. NC, control group; PC, with BBC; BF, bacteriophage in feed (10^8^ pfu /kg diet) + BBC; BN, bacteriophage nasal spray (2 × 10^7^ pfu) + BBC; AT, 1 g florfenicol/kg + BBC.

The concentration of plasma zonulin was increased in PC pigs compared with the NC (Fig. 2a). The BF treatment showed lower plasma zonulin compared with the BN and AT treatments. Similarly, compared with the NC, the fecal lipocalin was markedly increased in PC pigs (Fig. 2b). Compared with the NC pigs, plasma concentrations of malondialdehyde (MDA) were markedly increased in the PC pigs, whereas MDA concentration was significantly lower in AT pigs compared with the BF and BN (Fig. 2c). The plasma superoxide dismutase (SOD) levels were higher in NC pigs compared with all the BBC pigs. A higher plasma concentration of SOD was observed in the BF and AT treatments compared with PC and BN treatments (Fig. 2d). The concentration of lipopolysaccharide (LPS) and lipopolysaccharide-binding protein (LBP) in the enterocytes was increased in PC pigs compared with the other treatments (Fig. 2e). The BF and BN treatments showed higher intestinal LPS compared with the AT treatment, however, no difference was detected in the concentration of LBP between the NC, BF, BN, and AT treatments.

### Pathways metabolomics

**Figure 3:**
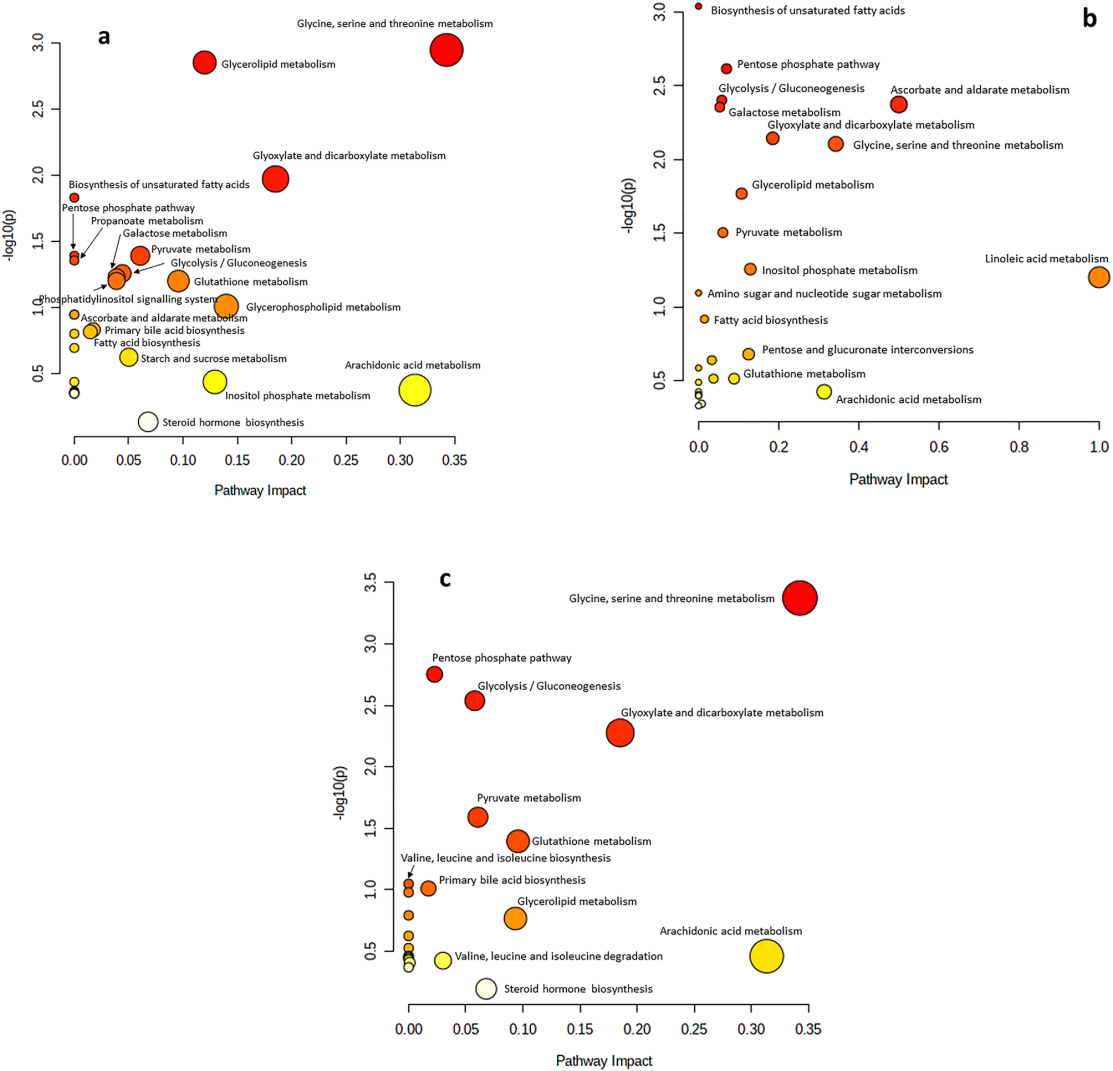
KEGG metabolic library analysis using metaboloanalyst. Based on the metabolite concentrations, the change between treatments. BN, bacteriophage nasal spray; and NC, without *B. bronchiseptica* challenge (**a**), BN, bacteriophage nasal spray (2 × 10^7^ pfu) and PC, positive control (with *Bordetella* challenge) (**b**), and BN, bacteriophage nasal spray (2 × 10^7^ pfu) and AT, 1 g florfenicol/kg + BBC (**c**), and their effects on the function of the pathway through changes in key pathway junction sites were evaluated. Results from all 30 pig routes are concurrently shown to show which ones are the most important in terms of effect and *Bordetella* challenge P-value.

The results of pathway analysis and enrichment analysis metabotype as generated by MetaboAnalyst 4.0 web-based software. Based upon the change in metabolites concentrations (Fig. 3), metabolic pathways analysis identified that the BN treatment mainly influenced the glycine, serine-threonine metabolism; glycerolipid metabolism; glyoxylate and dicarboxylate metabolism; and arachidonic acid metabolism compared with the NC treatment (Fig. 3a). The comparison between the BN and PC treatments showed the change in ascorbate and aldarate metabolism, linoleic acid metabolism, glycine, serine and threonine metabolism, biosynthesis of unsaturated fatty acids, pentose phosphate pathway, glycolysis/gluconeogenesis, galactose metabolism, glycerolipid metabolism, and glyoxylate and dicarboxylate metabolism, and arachidonic acid metabolism (Fig. 3b). The comparison between the BN and AT treatments showed the change in glycine, serine-threonine metabolism, glyoxylate-dicarboxylate metabolism, and arachidonic acid metabolism (Fig. 3c).

**Figure 4:**
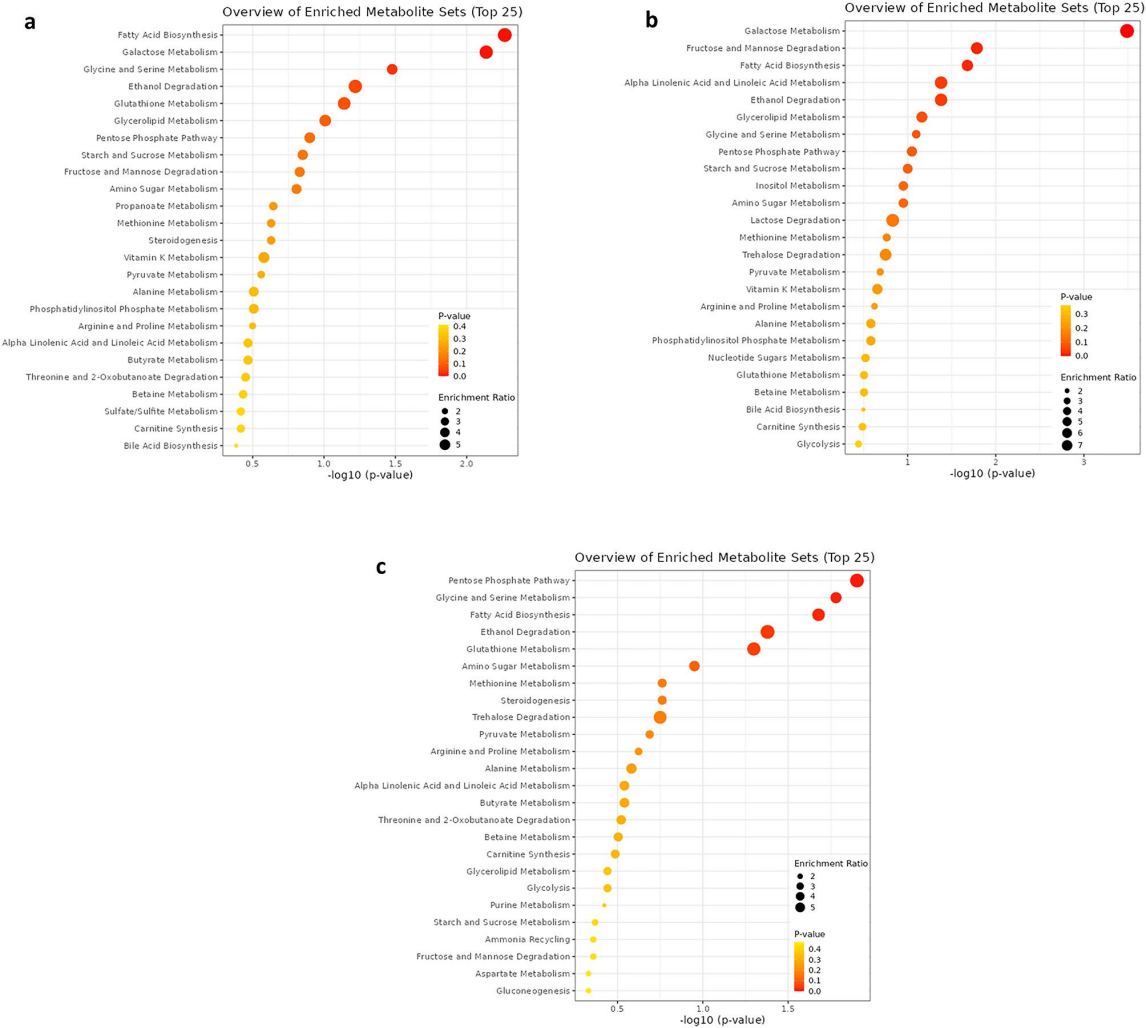
Based on the small molecule pathway database, a dot plot perspective shows the overview of the over-representation enrichment. Sets of pathway metabolites are arranged according to fold enrichment and P-value for the change between BN, bacteriophage nasal spray and NC negative control (without *B. bronchiseptica* challenge) (**a**), BN, bacteriophage nasal spray (2 × 10^7^ pfu) and PC, positive control (with *Bordetella* challenge) (**b**), and BN, bacteriophage nasal spray (2 × 10^7^ pfu) and AT, 1 g florfenicol/kg + BBC (**c**).

Figures 4 depict bar charts and dot plot views of the enriched metabolite sets. The pathway analysis of the enriched metabolite sets showed that fatty acid biosynthesis, galactose metabolism, and glycine-serin metabolism had the highest impact among 25 implicated metabolite sets in the BN treatment compared with the NC (Fig. 4a; *p* < 0.05 and FDR < 0.1), whereas galactose metabolism, fructose-mannose degradation, fatty acid biosynthesis, alpha-linolenic acid-linoleic acid metabolism, and ethanol degradation was enriched in BN pigs compared with the PC (Fig. 4b). The pentose phosphate pathway, glycine-serin metabolism, and fatty acid biosynthesis had the highest impact among the metabolites in the BN compared with the AT (Fig. 4c).

**Figure 5:**
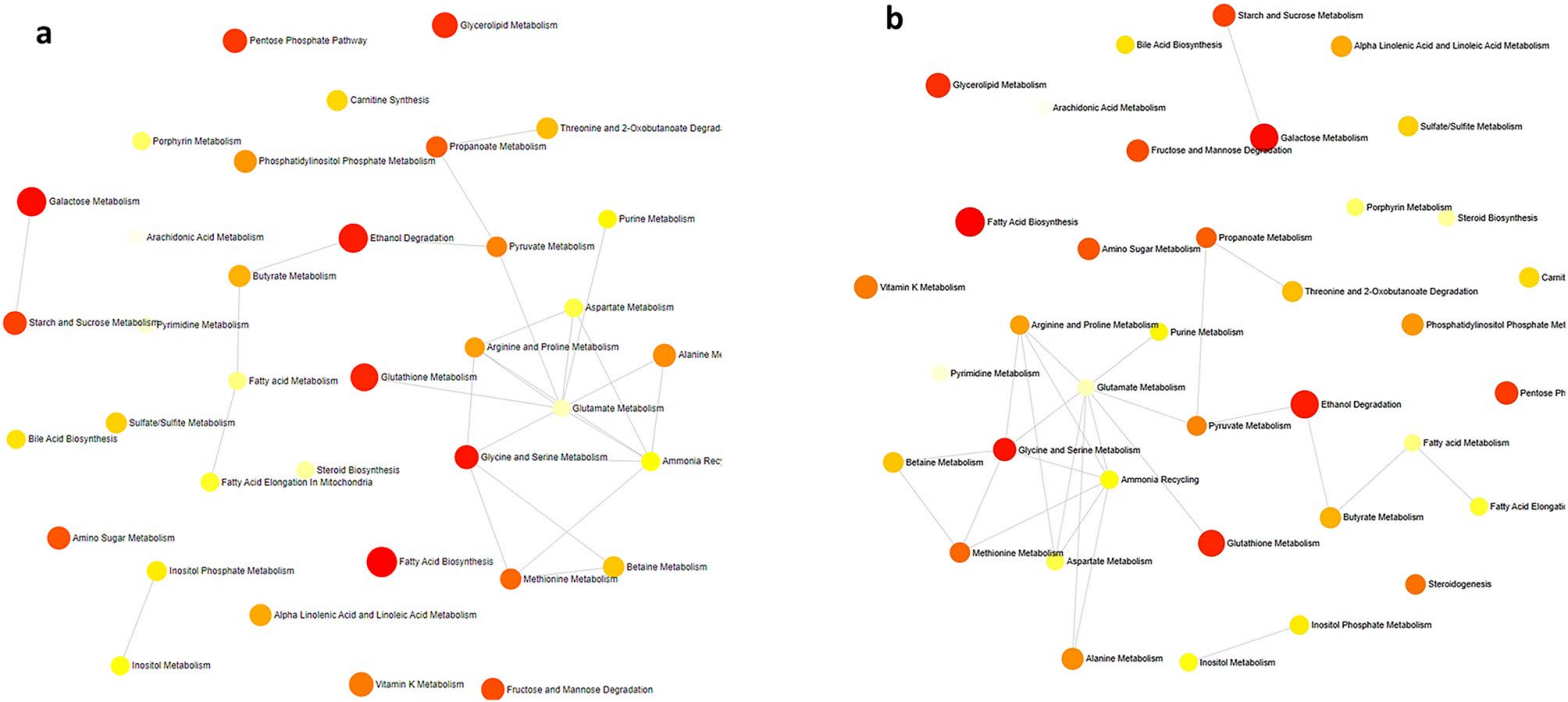
Investigation of metabolic biomarkers' pathway enrichment in BN therapy. The network diagram shows pathways that are closely associated with pig metabolic disorders between BN, bacteriophage nasal spray and NC, negative control (without *B. bronchiseptica* challenge) (**a**), BN, bacteriophage nasal spray (2 × 10^7^ pfu) and PC, positive control (with *Bordetella* challenge) (**b**). FDR-adjusted *p*-values for each of the shown metabolic pathways are less than 0.001. The show fold enrichment was shown by red hexagon sizes. False discovery rate, or FDR.

The main enriched metabolites were galactose metabolism, starch-sucrose metabolism, fatty acid biosynthesis, ethanol degradation, glycine-serin metabolism, and glutathione metabolism for BF treatment compared with the PC (Fig. 5a). Glycerolipid metabolism, galactose metabolism, starch-sucrose metabolism, ethanol degradation, glycine-serin metabolism, glutathione metabolism, and fatty acid biosynthesis in the BF treatment compared with the NC (Fig. 5b).

**Figure 6:**
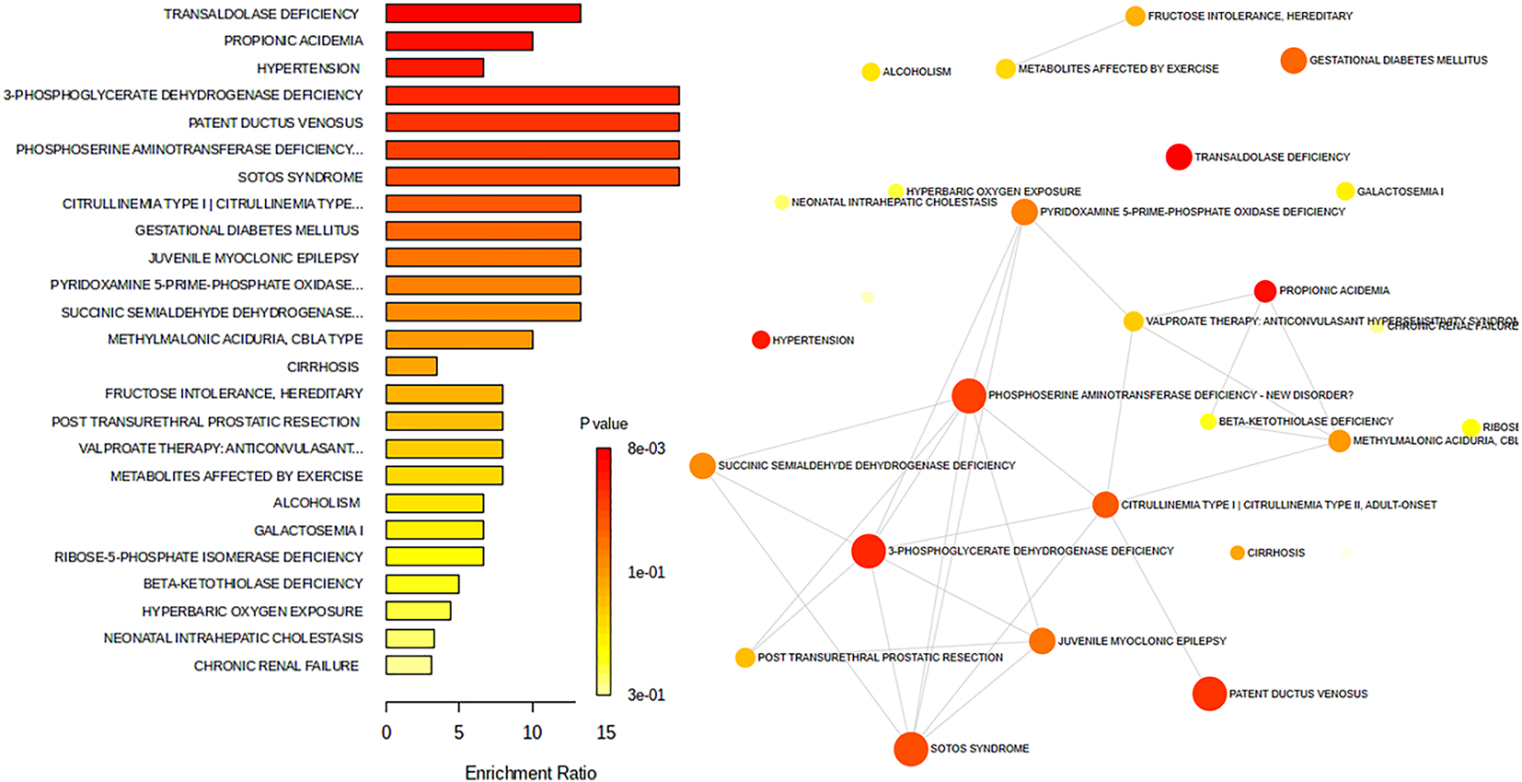
The comparison of disease-related signature enrichment overview based on 344 metabolite set between nasal spray bacteriophage in pigs and human blood using metaboanalyst. Based on the small molecule pathway database, a dot plot perspective shows the overview of the over-representation similarity between diseases.

The disease-related signature enrichment overview (Fig. 6) based on 344 metabolite sets reported in human blood using metaboanalyst showed a significant resemblance between the NC and BN treatments for transaldolase deficiency, propionic acidemia, hypertension, 3-phosphoglycerate dehydrogenase deficiency, patent ductus venosus, phosphoserine aminotransferase deficiency, and sotos syndrome.

## Discussion

*Bordetella* spp. respiratory infections are most commonly associated with pneumonia, a contagious respiratory illness that affects pigs^7^. One of the main symptoms of a *B. bronchiseptica* infection is inflammation of the turbinate, which are bony structures in the nasal passages that help to filter and humidify the air that we breathe^7^. Inflammation of the turbinates can cause swelling and congestion of the nasal passages, leading to difficulty breathing and a decrease in the turbinate score^25^. Our results showed that the use of bacteriophages is necessary to control the lesions in pigs. However, there was no difference between the BF and BN treatments, the BF pigs showed higher turbinate score compared with the NC, indicating the importance of using bacteriophages as a nasal spray rather than in the feed. The turbinate score is a measure of the size and function of the turbinates to assess the health of the respiratory system^12,26^. A higher turbinate score indicates that the turbinates are smaller or less functional, which can be a sign of respiratory illness or other health problems^26^. In diseased pigs, the turbinates may become swollen or inflamed, which can affect their function and lead to respiratory symptoms such as nasal discharge, sneezing, and difficulty breathing^7^. This condition is known as turbinate hypertrophy or turbinate hyperplasia. Our study confirmed that treatment for a *B. bronchiseptica* infection typically involves the use of antibiotics. The turbinate score was lower in the AT treatment compared with the PC and BF treatments showing that the use of bacteriophage in the diet is not as effective as antibiotics in controlling *B. bronchiseptica* infection.

The inflammation level was the highest in the PC treatment due to high blood IL-1β, IL-6, and TNF-α Levels. Inflammation is the major body response to infection and injury by sending immune cells to the affected area^1,3,6^. These immune cells release chemical signals that help to repair damaged tissue and fight off the invading pathogen^6^. In the case of *B. bronchiseptica* infection, the bacteria can cause inflammation in the respiratory tract, leading to symptoms such as coughing, sneezing, and difficulty breathing^2^. This study confirms that treating a *B. bronchiseptica* infection typically involves the use of antibiotics to kill the bacteria and reduce inflammation. IL-1β and TNF-α are cytokines, which are secreted by the immune system cells and play a crucial role in the immune response^3^. Both cytokines are involved in inflammation, and their production is enhanced in response to *B. bronchiseptica* infection or tissue injury^1,2^. IL-1β plays a crucial role in controlling infection and injury, but excessive and uncontrolled production can lead to chronic inflammation and contribute to the development of inflammation^6^. The high production of IL-1β and TNF-α occurred in the PC pigs, which is an indicator of high inflammation by the BBC. The secretion of IL-1β and TNF-α in the BN pigs was down-regulated compared with the BF pigs. IL-6 is also responsible for the acute phase of inflammation. The secretion of TNF-α, IL-1β, and IL-6 can be up-regulated in response to infection with *B. bronchiseptica*^1,2^*,* as these cytokines play a role in the immune response to the bacteria.

The severity of *Bordetella* spp. pneumonia can be increased by the proliferation of opportunistic bacteria such as *S. aureus*, *E. coli*, and *S. suis*^9–11,27^. Our study showed that *B. bronchiseptica* infection increases the risk of opportunistic bacteria colonization in the lungs. The AT treatment showed the greatest result in controlling opportunistic bacteria because the used bacteriophage in our study was designed to lyse only *B. bronchiseptica* rather than *S. aureus*, *E. coli*, and *S. suis*. However, the population of *S. aureus* was reduced in the BF and BP treatment possibly due to the lower *B. bronchiseptica* damage in the turbinate, which resulted in a lower opportunity for bacterial growth. This could occur if the *B. bronchiseptica* infection weakens the respiratory system health, making it more susceptible to other infections. Our results confirmed that if *B. bronchiseptica* infection is left untreated, it can lead to severe pneumonia, which could potentially allow opportunistic bacteria to be colonized in the lungs. Opportunistic infections occur when the immune system is compromised^3,9,28^. *S. suis*, *S. aureus*, and *E. coli* are types of bacteria that can cause opportunistic infections in the lung^10,11,28,29^. *S. aureus* is found on the skin in the respiratory tract, and it can cause infections such as pneumonia, bronchitis, and sinusitis^9,30^. However, *E. coli* is commonly found in the digestive tract and can cause infections such as diarrhea and colibacillosis^31–33^, it can be found in the respiratory system during infection^8^. Bacteriophages were unable to control *S. suis* population, which is one of the main opportunistic bacteria that cause infections in the lung, such as respiratory tract infections and wound infections^27,28^. It is known that bacteriophages reduce the *Bordetella* spp. infection in different ways including lysing the bacteria by targeting virulence *B. bronchiseptica* genes, competition for nutrients, and inducing an immune response against the bacteria^6,27,34^. Bacteriophages can be considered a promising option to control *B. bronchiseptica* infections. However, the differences between the AT and BF or BN treatments show that more research is needed to fully understand their potential and how they can be effectively utilized in a clinical setting of *Bordetella* spp. infection and relative opportunistic bacteria colonization in the lungs.

Respiratory infections in pigs can also slightly lead to gastrointestinal symptoms such as diarrhea by stimulating the immune system and damaging the intestinal epithelial cells, resulting in increased gut permeability and inflammation^3,11^. The dramatic reduction of plasma zonulin concentration in the BF treatment shows the beneficial effects of bacteriophages in controlling the population of transferred *B. bronchiseptica* into the gasterointestinal tract. Zonulin is a protein that regulates the permeability of tight junctions in the gut lining^35,36^. Elevated levels of zonulin have been associated with increased gut permeability^35,37^, which is a hallmark of inflammatory diseases. The measurement of plasma zonulin levels is used as a marker of gut permeability and is being studied as a potential diagnostic tool for various diseases^35^. Fecal lipocalin-2 was reduced in the BF, BN, and AT treatments compared with the PC. Lipocalin-2 is a protein that is produced by intestinal epithelial cells and involved in regulating gut permeability and inflammation^35^. Elevated levels of fecal lipocalinrep-2 have been associated with increased gut permeability and inflammation, and are being studied as a potential biomarker for various gastrointestinal disorders.

The BBC increased the concentrations of MDA and decreased the concentration of SOD. SOD is an enzyme that catalyzes the conversion of superoxide into hydrogen peroxide, which can then be further converted into water by other enzymes like catalase^38^. By reducing the levels of superoxide, SOD helps to protect the body from oxidative damage^32^. The treatment process of injuries and superoxide production during the BBC can be responsible for the higher consumption of SOD^39^. The supplementation of bacteriophage improved the antioxidant status by increasing SOD compared with the PC. The increased ascorbate and aldarate metabolism pathway in the BN treatment produce vitamin C as an antioxidant factor that may be associated with SOD production. MDA, on the other hand, is a measure of lipid peroxidation, a process by which reactive oxygen species attack cellular lipids and cause oxidative damage^38,40^ and is considered a reliable marker of oxidative stress^41^. The concentration of MDA was reduced in the BN and BF treatments compared with the PC. Within the circulatory system, alterations in SOD and MDA concentrations serve as indicators of the occurrence of oxidative stress^40^, a phenomenon linked to respiratory tract issues and inflammation. Thus, the measurement of SOD and MDA levels in the blood may be used as biomarkers for infection stress.

Comprehensive metabolomics provides information on body metabolic state and how that status varies in various environmental circumstances^20,42^. Therefore, the enriched metabolism pathways in the PC, BN, and AT treatments may give further information about pigs requirements, which can be supplemented in their diets to reduce the adverse side effects of *B. bronchiseptica* infection. There is limited information on the effects of *B. bronchiseptica* on blood metabolites and pathways. However, it is known that infections can cause inflammation in the respiratory tract with effects on changes in blood metabolism^21,24,43^. In particular, infection with *B. bronchiseptica* may lead to an up-regulated secretion of proinflammatory IL-6, which can affect metabolism and energy production in the body^1,5^. The relationship between blood metabolomics, the immune system, and feed requirement is complex and requires further research to fully understand.

The amino acids including glycine, serin, and threonine metabolism were the most enriched pathways in the BN pigs compared with the NC and AT. It may emphasize the importance of dietary glycine, serine, and threonine content in the BN pigs. Glycine, serine, and threonine are all amino acids that are involved in several important metabolic pathways^43–45^, which can be enriched during sepsis in pigs^21^. Glycine and serine are dispensable amino acids, signifying that the organism has the capacity to endogenously synthesize them from other nutritional precursors, obviating the necessity for their direct acquisition from dietary sources^43,45^. Glycine is an important amino acid for the synthesis of proteins, and it is also involved in the synthesis of serotonin and gamma-aminobutyric acid, which are important for proper brain function^45^. Serine is also involved in protein synthesis, and it is also a substance for the production of certain lipids and the metabolism of folate^43^. Moreover, threonine is an essential component of proteins, and it is also included in the production of certain enzymes and hormones^44^. All these three amino acids can be obtained directly from the diet through the use of protein-rich diets, which shows the importance of higher dietary protein requirements during *Bordetella* spp. disease.

The ascorbate and aldarate pathway were enriched in the BN treatment compared with the PC. Vitamin C, commonly known as ascorbate, is a vital component needed for the body to operate properly during stressful conditions^46^. It is involved in several important metabolic pathways, including the synthesis of collagen, which is an essential protein for the formation of skin, blood vessels, and connective tissue^47^. Ascorbate is also important for the synthesis of neurotransmitters, such as norepinephrine and dopamine, and for the metabolism of iron^46,47^. Moreover, aldarate is a type of sugar that is involved in the metabolism of carbohydrates^18,20^. It is produced during the breakdown of carbohydrates, and it is also involved in the synthesis of certain lipids and the metabolism of certain amino acids^20^. Ascorbate and aldarate are both important for maintaining the overall health and functioning of the pigs.

The greater glycerolipid metabolism in the BN treatment rather than the NC can be due to the cellular injuries in the lung of the BBC pigs in order to treat the damaged cells. Glycerolipids are a type of lipid molecule that are composed of glycerol and one or more fatty acids with important roles in energy metabolism and membrane synthesis^23,48^. There are two main types of glycerolipids including glycerides and phospholipids^23,48^. Glycerides are the initial form of maintained energy and are synthesized in the liver and adipose tissue to form cell membranes^23^. Glycerolipid metabolism involves the synthesis, degradation, and interconversion of glycerides and phospholipids molecules^48^. Synthesis of glycerolipids occurs in the endoplasmic reticulum and involves the transfer of fatty acids to glycerol by the enzyme acyltransferase^23,48^. Degradation of glycerolipids occurs in the liver and other tissues through the action of lipases, which hydrolyze the ester bonds to release free fatty acids and glycerol^48^. Interconversion of glycerolipids can also occur through the action of various enzymes, such as phospholipase and glycerol kinase^23^. Overall, as the treated pigs in the BN and AT treatments showed overall metabolic improvement compared with the PC, it can be concluded that pigs overall health status the specific effects of *B. bronchiseptica* on blood metabolites and pathways may depend on the severity of infection and efficiency of treatment.

The metabolic pathways showed transaldolase and propionyl-CoA carboxylase deficiencies in the BN treatment compared with the NC metabolic pathways, which is a disorder that leads to elevated levels of certain sugars, such as sedoheptulose and erythrulose, in the blood and urine^18,21^. Impairment of the pentose phosphate pathway is important for the production of energy and the reduction of oxidative stress^20^. The compromised feed intake and carbohydrate intake in the BN treatment compared with the NC may be responsible for this result. Propionic acidemia involves a low-protein diet, which indicates the low efficiency of dietary protein^18,49^. Therefore, it can be concluded that the higher dietary protein may be beneficial during *the* BBC in order to enrich the BN pathways. The metabolic pathways of the BN treatment showed similarities to hypertension, which can be related to inflammation. Impaired nitric oxide signaling can cause hypertension through oxidative stress and inflammation^50^. Disorders of phospholipid and lipid metabolism are frequently observed during hypertension disease^50^, which may be in line with the glycerolipid metabolism in the BN treatment. We expected a different result but we found that there is not enough research on the effect of the respiratory tract on metabolite change. However, the exact role and similarity of these metabolites on the immune response in the BBD, and the effects of bacteriophages are not totally clarified and are the subject of ongoing research.

The severity of the infection and the resulting weight loss depends on age and immune status of the pig, the virulence of the strain of *B. bronchiseptica*, and the presence of other infections or underlying health conditions^4,5,12^. In general, pigs after weaning tend to be more susceptible to infections and may experience more severe symptoms^15,31,51^. The choice of young weaned pigs in the current study can be one of the reasons responsible for such significant differences between the treatments. Treatment for *B. bronchiseptica* infections in pigs typically involves the use of antibiotics, as well as supportive care such as providing the pigs with a clean environment^12^. In agreement, the supplementation of antibiotics increased pigs health compared with the BF treatment but no difference was shown in comparison to the BN treatment. The result of the current study shows that the use of bacteriophages as the nasal spray is more effective than using in diet. It can be because of the lower number of available bacteriophages in the lung in the BF treatment.

## Conclusion

In summary, our study revealed significant reductions in turbinate scores and the secretion of proinflammatory cytokines, including IL-1β, IL-6, and TNF-α, in response to treatments involving bacteriophage when compared to the PC. Notably, the application of nasal spray bacteriophage led to metabolic alterations, enriching pathways related to ascorbate and aldarate metabolism, glycine, serine, and threonine metabolism, as well as linoleic acid metabolism, in contrast to the PC treatment. Our findings suggest that the utilization of bacteriophages via nasal spray delivery holds promise in mitigating inflammatory responses, particularly IL-1β, IL-6, and TNF-α, compared to their dietary administration. The nasally sprayed bacteriophage showed potential as a superior therapeutic approach for reducing inflammation. However, it is essential to acknowledge the valuable anti-*B. bronchiseptica* effects demonstrated by dietary bacteriophages, which make them a practical choice for *B. bronchiseptica* control, especially in cost-effective large-scale pig farming operations. Further investigations may be conducted to assess the efficacy of higher doses of dietary bacteriophages. Our results not only provide insights into how pig metabolism can be influenced by bacteriophages and antibiotics during *B. bronchiseptica* challenge, but they also offer foundational knowledge for enhancing pig diets based on their specific metabolic requirements. These findings underscore the potential benefits of dietary supplementation with glycine, serine, threonine, linoleic acid, and vitamin C in optimizing pig health and productivity in the context of *B. bronchiseptica* control.

## Material and methods

### Study design

A total of 30 (24-day-old) weaned piglets (Landrace × Yorkshire × Duroc; average weight of 7.57 ± 0.32 kg) were subjected to be distributed between 5 treatments with 6 replicates in separated pens. Fecal and nasal samples were collected at the beginning of the experiment to confirm piglets were *B. bronchiseptica* and *Salmonella* free. The treatments included: NC, without challenge; PC, BBC; BF, 10^8^ pfu bacteriophage/kg diet and BBC; BN, 2 × 10^7^ pfu/day bacteriophage by nasal spray and BBC; AT, antibiotic and BBC. The experiment was conducted for 2 weeks. The diets were formulated to provide all of the nutrients to meet the nutrient requirements recommended by NRC^52^. After 3-d adaptation in order to ensure controlling post-weaning diarrhea, the *B. bronchiseptica* challenge (2 × 10^7^ CFU) was performed at d 0 on 24-day-old piglets by the nasal spray. Portal vein were punctured to obtain blood samples (8 mL) at d 1, 3, 7, and 14, which were then put in vacuum tubes (5 mL) and heparinized tubes (5 mL) for analysis^53^. To separate the serum samples, the samples were centrifuged at 3000 rpm for 15 min at 4 °C in vacuum tubes. The serum samples were then kept at − 80 °C for further analysis. The fecal samples from each sow were collected, frozen, and pooled, dried for 72 h in a forced-draft oven (65 °C), crushed through a 1-mm screen, and well mixed, and then a subsample was obtained for chemical analysis.

### *B. bronchiseptica* preparation

*B. bronchiseptica* strains (BB-01) were cultured and kept on Brain Heart Infusion (BHI) agar (Difco; Cat. No. 241830). To cultivate the bacteria to mid-log phase, they were cultured for 15 h at 37 °C in a shaking incubator using BHI broth (Bacto; Cat. No. 237500). The serial dilution and subsequent colony counting were performed to determine bacterial concentration. The final concentration was assessed by measuring the optical density at 600 nm (OD600) and then adjusted to 2 × 10^8^ colony-forming units per milliliter (cfu/ml). Prior to their use in experiments, *B. bronchiseptica* was subjected to two rounds of washing in phosphate-buffered saline (PBS) by centrifuging at 3500 × g for 6 min. The bacteria were then re-suspended in PBS to eliminate any residual materials from the growth medium.

### Bacteriophage

The Podoviridae bacteriophage Bor-BRP-1 (Accession number: KCTC 12705BP) was isolated from nature and was targeted to eliminate *B. Bronchiseptica* (Patent number: 10894068; Intron Biotechnology, Inc; Jan 19, 2021; International Classification: A61K 35/76 (20150101); C12N 7/00 (20060101); A61P 31/04 (20060101). The source of these bacteriophages was the sewage and excreta of swine farm in Korea. The bacteriophages were isolated through a series of steps, including plaque isolation, cultivation with host strains, purification, and filtration. The focus was on selecting bacteriophages with bacteriolytic activity against *B. Bronchiseptica*. The selected bacteriophage was manufactured using an individual bacteriophage powder, as recommended by Lee et al.^54^. The process involved co-cultivating the bacteriophage with a log-phase host strain in tryptic soy broth (Detroit, MI, USA) at 37 °C until host bacterial cell lysis was observed. The culture was then centrifuged (10,000 g; 4 °C; 30 min) and filtered (0.45 m syringe filter) to enhance the bacteriophage titer. To increase the bacteriophage titer, the method was performed twice, from co-cultivation to filtering. After mixing Maltodextrin (50%, w/v), the obtained filtrates containing individual bacteriophages were freeze-dried and then ground in a mill. After being combined with commercial complete feed at a weight ratio of 0.1%, or using as nasal spray 10^8^ pfu/kg the bacteriophage was given to pigs. Plaque-forming units per gram (pfu/g) for bacteriophage (Bor-BRP-1) species were modified to be about 10^8^ in the bacteriophage product.

### Turbinate score

All infected pigs were slaughtered at the end of experiment (day 14) to assess the macroscopic lung lesions and turbinate alterations that occurred during the BBC challenge. Each animal necropsy was performed right after euthanasia, with a focus on the respiratory system (lung score, turbinate score). Each pig's turbinate score was determined following necropsy using the techniques outlined by Gatlin et al.^26^. The upper first premolar tooth is where the snouts were divided in half. The grade for typical turbinates was 0. Grade 1 showed slight but noticeable atrophy. Grade 2 was assigned to moderate atrophy (not less than 50% of the turbinates). The dorsal and ventral scrolls had severe atrophy, which was given a grade of 3–6. Each pig's total visual turbinate scores, which ranged from 0 to 6, were then determined from the left and right turbinates and septum.

### Microbial test

Bronchoalveolar lavage fluid samples of all pigs from five treatments were collected at d 14 of experiment. To ensure the maximum possible DNA concentration in the final eluate, 1 mL of inhibitEX buffer was added to each stool sample and vortexed continuously for 1 min. The homogenized material was vortexed for 15 s while being heated in a water bath at 70 °C for 5 min to ensure homogeneous lysis. After that, samples were centrifuged for 1 min at maximum speed (20,000 × g, 14,000 rpm) to pellet particles. 600 µL of the step 1 supernatant was pipetted into the 2 mL microcentrifuge tube containing proteinase K, and 25 µL of proteinase K was added to the tube. We added 600 µL of buffer AL and vortexed for 15 s to produce a homogenous solution, which was then incubated for 10 min at 70 °C before being quickly centrifuged to remove any droplets that had collected on the tube lid. The lysate was mixed with 600 µL of ethanol (96–100%) before being briefly centrifuged to remove any remaining droplets from the tube lid. A 600 µL of the aforementioned lysate was added to the QIAamp spin column and spun for 1 min at maximum speed (20,000 × g, 14,000 rpm). The old tube containing the filtrate was removed, and a fresh 2 mL collection tube was used to hold the QIAamp spin column. After carefully opening the QIAamp spin column, 500 µL of buffer guanidinium chloride was introduced, and the centrifuge was run at full speed for 1 min. The previous tube containing the filtrate was discarded and a fresh 2 mL collection tube was used to transfer the QIAamp spin column. A second time, the QIAamp spin was gently opened, 500 µL of buffer AW2 tris-based ethanol solution was added, and it was centrifuged for three minutes at full speed. Insert the QIAamp spin column in a fresh 2 mL collection tube and discard the previous one that contained the filtrate. For three minutes, centrifuge at maximum speed to obliterate any potential buffer AW2 tris-based ethanol solution carryover. The QIAamp spin column was then transferred into a fresh, labeled 1.5 mL microcentrifuge tube, and 200 µL buffer ATE was pipetted directly onto the QIAamp membrane. This was followed by 1 min of room temperature incubation, 1 min of full-speed centrifugation, and 1 min of DNA elution. The DNA was then checked using a spectrophotometer. In our experiment, 10 ng of DNA, 1 × universal SsoAdvancedTM universal SYBR® Green Supermix, 2.5 ng/μL of each forward and reverse primer of *Escherichia coli*, *Streptococcus suis, Staphylococcus aureus,* and *B. bronchiseptica* (Table 3). After the PCR amplification step, a melting curve analysis was conducted. The PCR cycling parameters involved initial enzyme activation at 95 °C, followed by 40 cycles of denaturation at 95 °C for 15 s, annealing at the specified times and temperatures according to each primer, and extension at 72 °C for a specified time according to each primer^55,56^. The SYBR thermal cycling protocol was adhered to as recommended. The melting curve analysis provided information about the specificity of the amplification, ensuring the absence of non-specific products. The data from the melting curve analysis were analyzed using the Rotor-gene Q 2plex software (Corbett Life Science Qiagen 2008) to confirm the specificity of the PCR products.

**Table 3:** Genes and primer sequences used for real-time PCR.

Strain		Sequence(5′-3′)
*β-Actin*	AJ312193	CTCCTTCCTGGGCATGGA
		CGCACTTCATGATCGAGTTGA
*Escherichia coli*	uidA	AAAACGGCAAGAAAAAGCAG
		GCGTGGTTACAGTCTTGCG
*Streptococcus suis*	gdh	GCAGCGTATTCTGTCAAACG
		CCATGGACAGATAAAGATGG
*Staphylococcus aureus*		AATCTTTGTCGGTACACGATATTCTTCACG
		CGTAATGAGATTTCAGTAGATAATACAACA
*Bordetella bronchiseptica*	fla	TGGCGCCTGCCCTATC
		AGGCTCCCAAGAGAGAAA

### Plasma, intestine, and feces samples

The technique published by Ha et al.^40^ was used to measure plasma SOD. Utilizing a previously described indirect competition test between SOD and the indicator chemical, nitroblue tetrazolium, for superoxide produced by xanthine-xanthine oxidase, total SOD activity was calculated. By defining 1 unit of SOD activity as the quantity of sample protein capable of blocking nitroblue tetrazolium reduction by 50% of maximal inhibition, SOD activity units were computed. The information was expressed as U/mg protein and adjusted to the sample's protein content. According to Hosseindoust et al.^38^, the reaction of MDA and thiobarbituric acid (TBA) was used to measure plasma lipid peroxidation. Briefly, in a boiling tube, 1 mL of thawed plasma sample was mixed with 1 mL of ethylenediaminetetraacetic acid (0.037 g EDTA in 10 mL distilled water), 2 mL of trichloroacetic acid (3 g trichloroacetic acid in 30 mL distilled water), and 1 mL of butylated hydroxytoluene (0.2 g After that, the mixture was centrifuged for 15 min at 1200 g. After that, 1 mL of the supernatant was incubated for 20 min at 90 °C with 1 mL of TBA (0.134 g TBA in 20 mL distilled water). After cooling, the absorbance at 532 nm wavelength was measured using a spectrophotometer (Bausch and Lomb Supertonic 70, Feldkirchen, Germany). According to Moturi et al.^35^, serum and fecal samples were examined for LPS, zonulin, lipocalin-2, and LBP (Nanjing Jiancheng Bioengineering Institute, Nanjing, China) using an enzyme-linked immunosorbent assay technique (ELISA). Sandwich enzyme-linked immunosorbent test was used to quantitatively assess circulating cytokines in serum (Thermo Fisher Scientific, Waltham, MA, USA). Commercial ELISA kits (MyBioSource, San Diego, CA, USA) of IL-1β (MBS2702037), IL-6 (MBS2701081), and TNF-α (MBS761006) were purchased. The manufacturers' recommended guidelines were followed for every detection. According to the standard curves created separately in each experiment, the cytokine concentrations were determined. Every assay was run twice, and each time, duplicates of each sample were evaluated.

### Pathways-metabolomics

The concentration of metabolites in pigs blood was determined using GC–MS. 100 µg plasma sample was placed in 5 mL centrifuge tubes, diluted with 500 μL water, and vortexed for 60 s, according to Moturi et al.^35^. Then, 1000 μL methanol was considered as an internal control to be added and vortexed for 30 s. The ultrasonic machine was applied to keep samples at 25 °C for 10 min after 30 min of incubation on ice. The centrifuge treatment (5000 rpm; 5 °C; 15 min) was then completed. After being dried, all of the supernatants were collected in 2 mL centrifuge tubes. Following the addition of the dried materials, 60 L of methoxyamine solution in pyridine was added, vortexed for 30 s, and then the mixture was allowed to react for 120 min at 37 °C. 90 min at 37 °C were spent centrifuging 60 L of trifluoroacetamide reagent (containing 1% FMCS) at 5000 rpm, 5 °C, and 15 min. Using an Agilent 7890A/5975C GC–MS, the resulting supernatant was transferred to a sample vial and examined (Agilent Technologies, Santa Clara, CA, USA).

### Statistical analysis

The GLM procedure was used to do the statistical analysis (SAS Inst. Inc. Cary, NC). Pigs in pen and initial BW were considered as fixed factors in the statistical model. Tukey multiple range tests were used to distinguish the treatment means. The experimental unit for each variable's investigation was an individual pig. Values of probability 0.05 were regarded as significant. To evaluate the metabolites, the raw data were processed, the metabolites were found, and they were normalized to (13C2)-myristic acid and stable isotope IS (http://srdata.nist.gov/gateway, accessed on 6 January 2023). The software program SIMCA-P + version 13.0 was used to carry out the statistical analysis (Umetrics, Umea, Sweden). The metabolites that could be assessed in relation to the *B. bronchiseptica* challenge, bacteriophage and antibiotic consumption were those with Variable Importance in Projection values of 1.0 and *p*-values of 0.05. The impact of BBC challenge and bacteriophage supplementation on metabolite set enrichment and metabolic pathways analyses were evaluated according to the online tool (http://www.metaboanalyst.ca/faces/ModuleView.xhtml, accessed on 6 January 2023)^19^.

### Ethics approval

The institutional animal care and use committee at Kangwon National University in Chuncheon, Republic of Korea, approved the experimental procedures in accordance with the Republic of Korea's regulations for the management of affairs involving experimental animals (KW-210503-6) and ARRIVE guidelines.

## Data Availability

The datasets generated during and/or analyzed during the current study are available from the corresponding author upon reasonable request.
